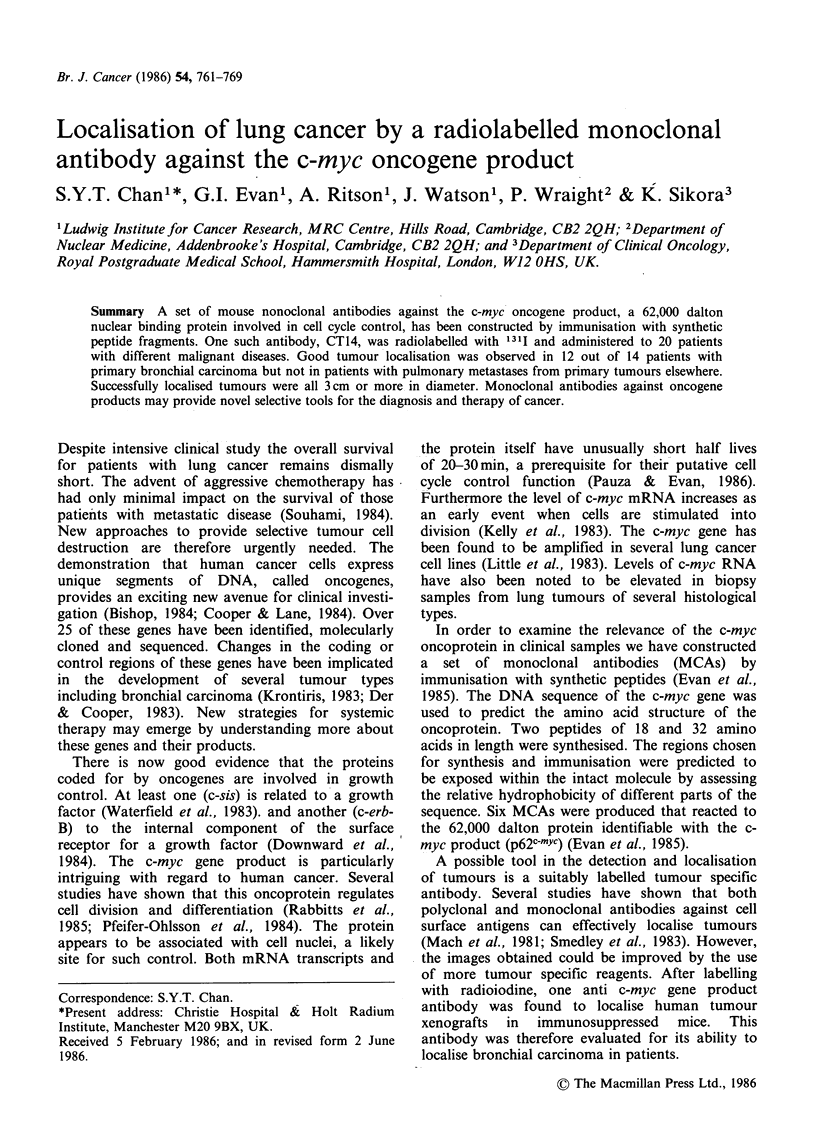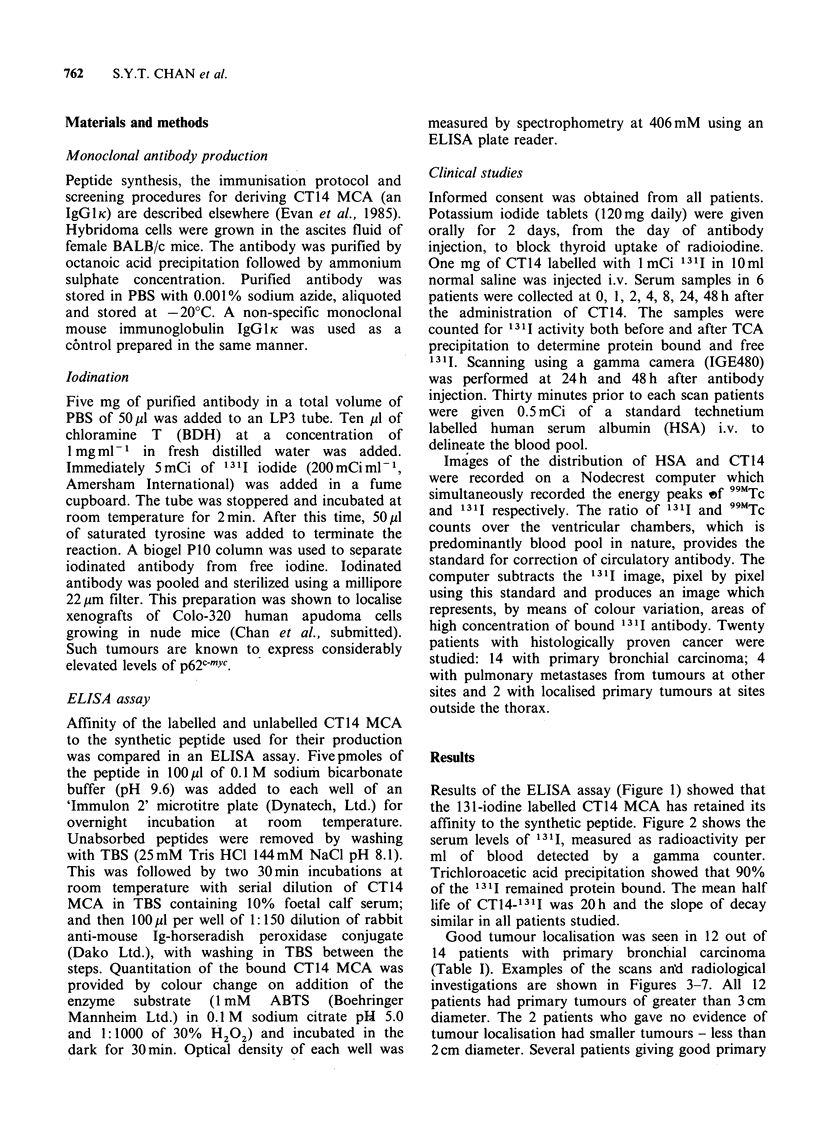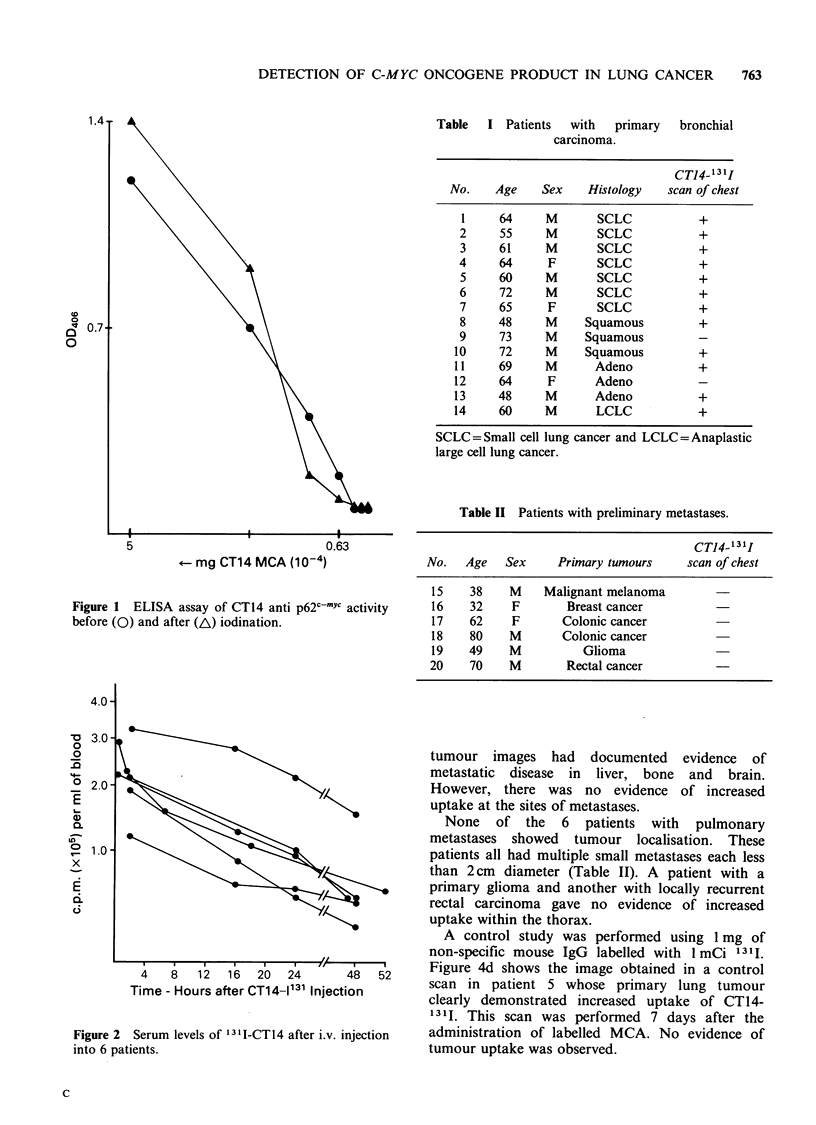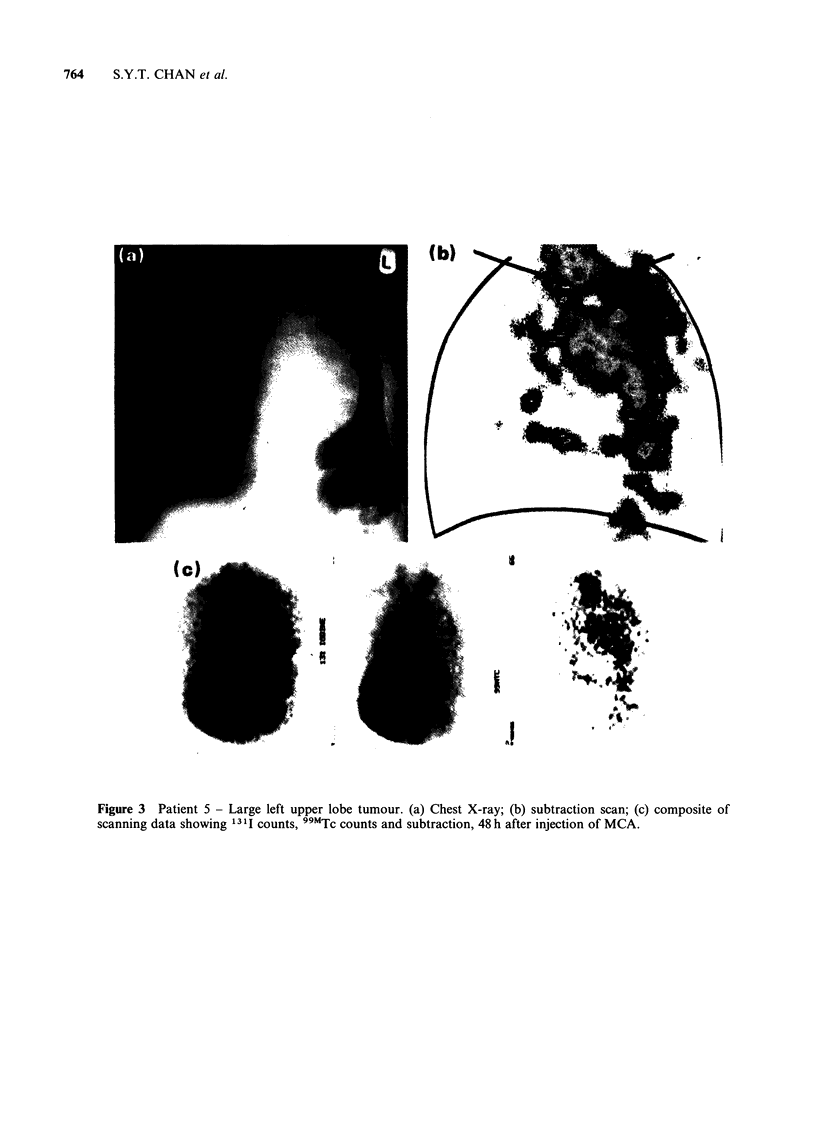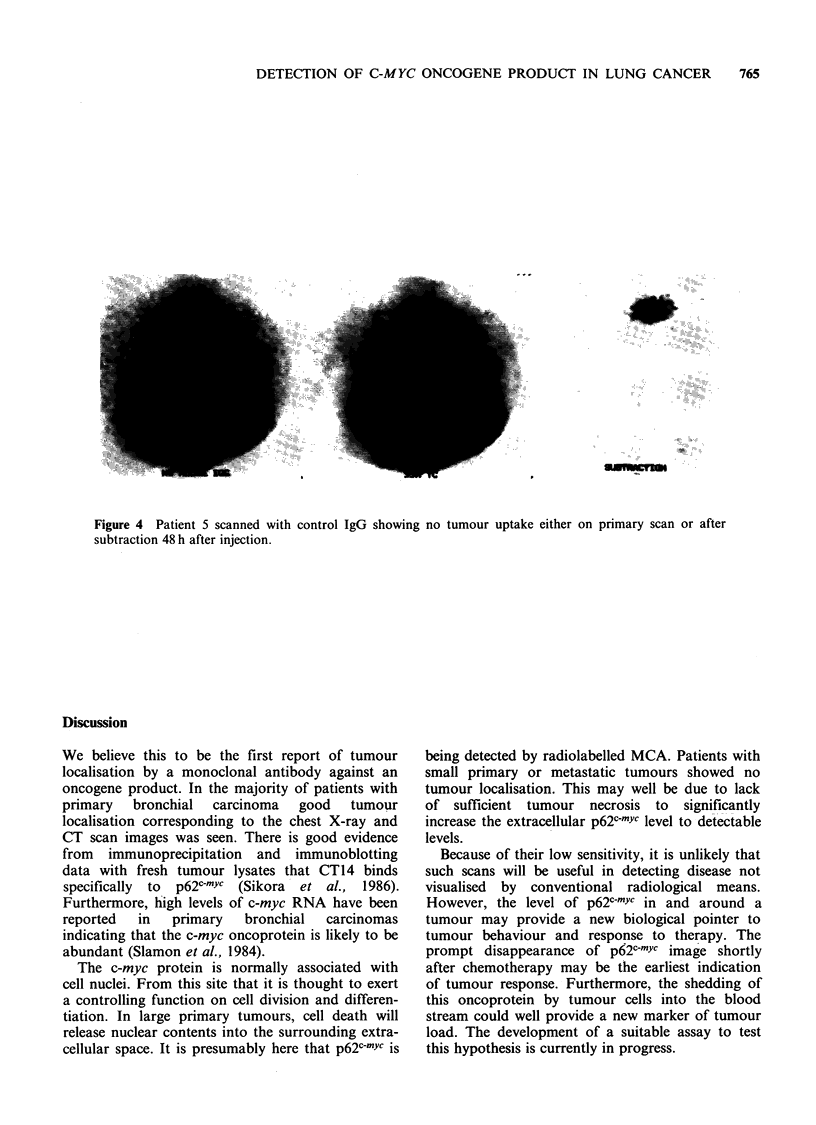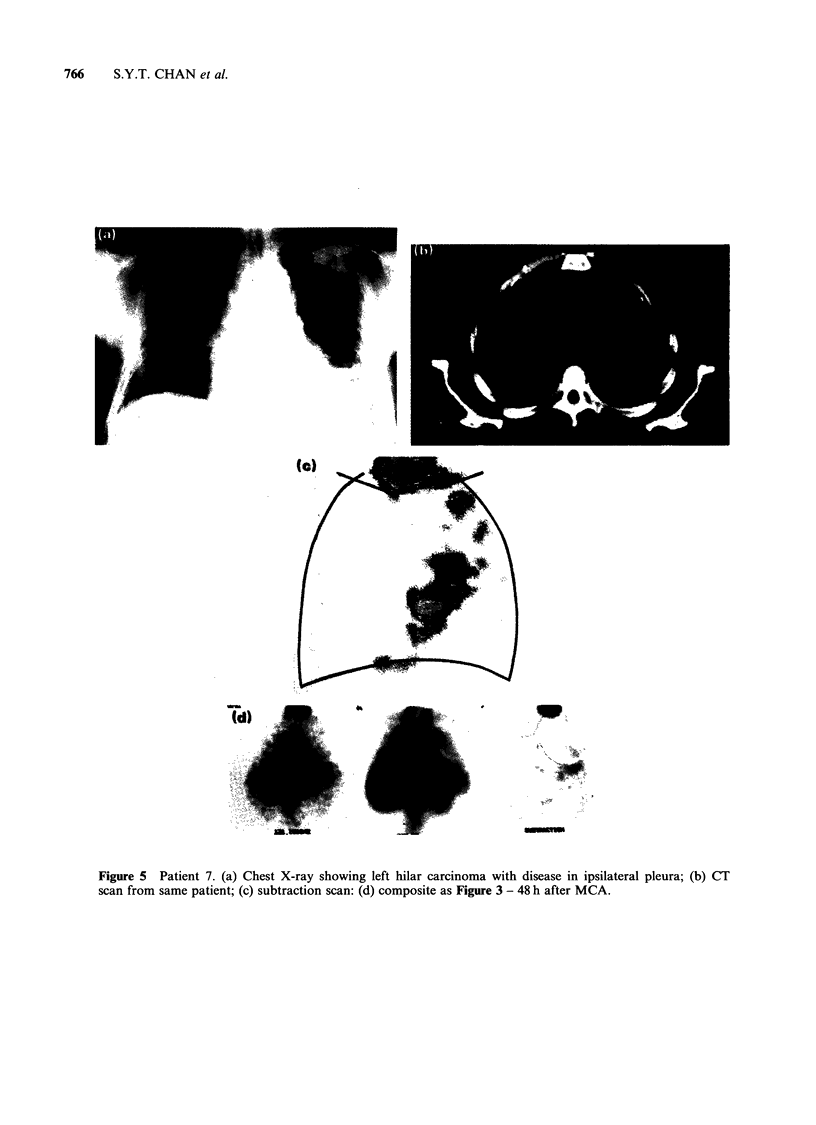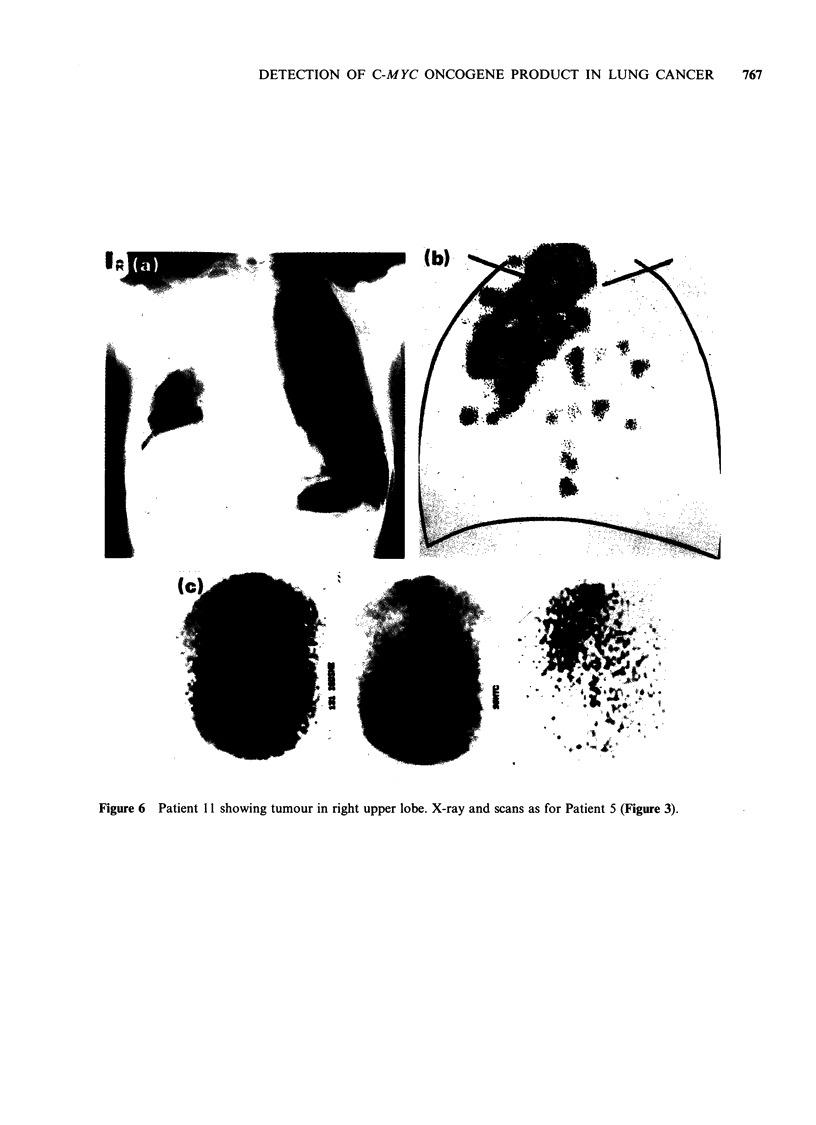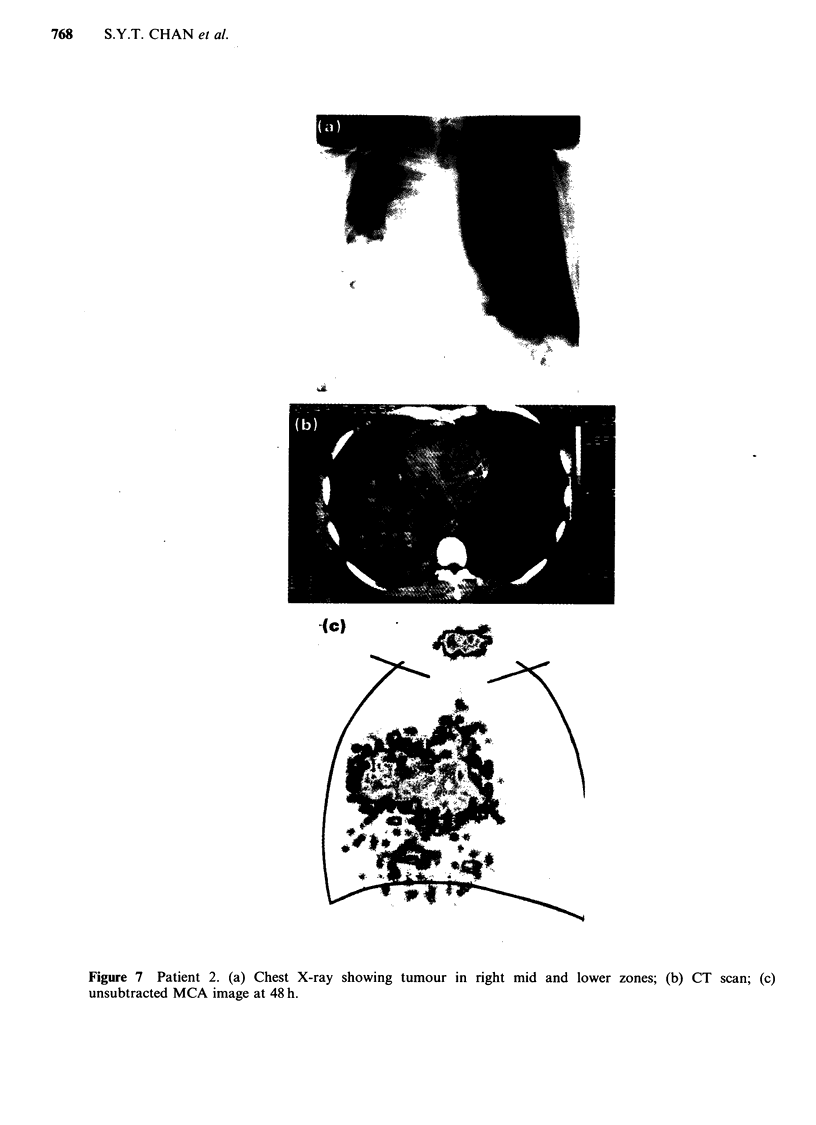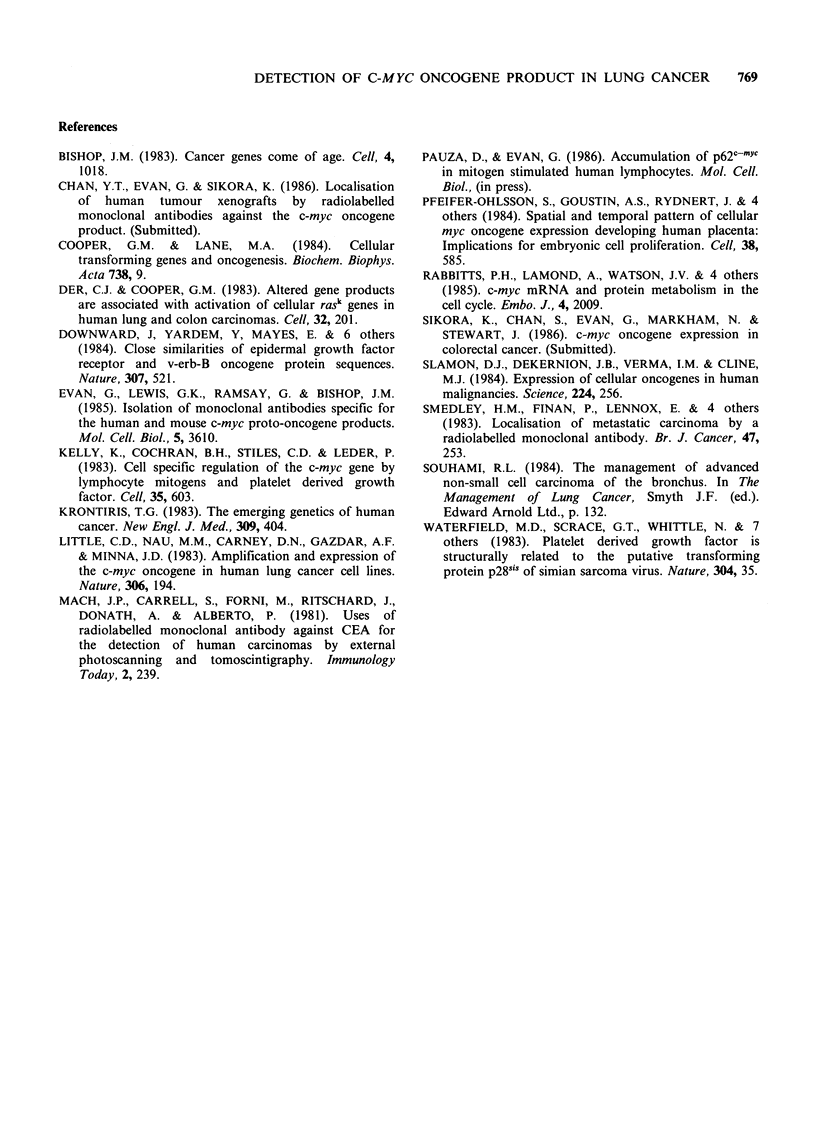# Localisation of lung cancer by a radiolabelled monoclonal antibody against the c-myc oncogene product.

**DOI:** 10.1038/bjc.1986.238

**Published:** 1986-11

**Authors:** S. Y. Chan, G. I. Evan, A. Ritson, J. Watson, P. Wraight, K. Sikora

## Abstract

**Images:**


*Br. J. Cancer* (1986) **54**, 761–769

### Localisation of lung cancer by a radiolabelled monoclonal antibody against the c-*myc* oncogene product

S.Y.T. Chan[1]*, G.I. Evan[1], A. Ritson[1], J. Watson[1], P. Wraight[2] & K. Sikora[3]

[1]*Ludwig Institute for Cancer Research, MRC Centre, Hills Road, Cambridge, CB2 2QH;* [2]*Department of Nuclear Medicine, Addenbrooke's Hospital, Cambridge, CB2 2QH; and* [3]*Department of Clinical Oncology, Royal Postgraduate Medical School, Hammersmith Hospital, London, W12 0HS, UK.*

**Summary** A set of mouse nonoclonal antibodies against the c-*myc* oncogene product, a 62,000 dalton nuclear binding protein involved in cell cycle control, has been constructed by immunisation with synthetic peptide fragments. One such antibody, CT14, was radiolabelled with $^{131}I$ and administered to 20 patients with different malignant diseases. Good tumour localisation was observed in 12 out of 14 patients with primary bronchial carcinoma but not in patients with pulmonary metastases from primary tumours elsewhere. Successfully localised tumours were all 3 cm or more in diameter. Monoclonal antibodies against oncogene products may provide novel selective tools for the diagnosis and therapy of cancer.

Despite intensive clinical study the overall survival for patients with lung cancer remains dismally short. The advent of aggressive chemotherapy has had only minimal impact on the survival of those patients with metastatic disease (Souhami, 1984). New approaches to provide selective tumour cell destruction are therefore urgently needed. The demonstration that human cancer cells express unique segments of DNA, called oncogenes, provides an exciting new avenue for clinical investigation (Bishop, 1984; Cooper & Lane, 1984). Over 25 of these genes have been identified, molecularly cloned and sequenced. Changes in the coding or control regions of these genes have been implicated in the development of several tumour types including bronchial carcinoma (Krontiris, 1983; Der & Cooper, 1983). New strategies for systemic therapy may emerge by understanding more about these genes and their products.

There is now good evidence that the proteins coded for by oncogenes are involved in growth control. At least one (c-*sis*) is related to a growth factor (Waterfield *et al.*, 1983). and another (c-*erb*-B) to the internal component of the surface receptor for a growth factor (Downward *et al.*, 1984). The c-*myc* gene product is particularly intriguing with regard to human cancer. Several studies have shown that this oncoprotein regulates cell division and differentiation (Rabbitts *et al.*, 1985; Pfeifer-Ohlsson *et al.*, 1984). The protein appears to be associated with cell nuclei, a likely site for such control. Both mRNA transcripts and the protein itself have unusually short half lives of 20–30 min, a prerequisite for their putative cell cycle control function (Pauza & Evan, 1986). Furthermore the level of c-*myc* mRNA increases as an early event when cells are stimulated into division (Kelly *et al.*, 1983). The c-*myc* gene has been found to be amplified in several lung cancer cell lines (Little *et al.*, 1983). Levels of c-*myc* RNA have also been noted to be elevated in biopsy samples from lung tumours of several histological types.

In order to examine the relevance of the c-*myc* oncoprotein in clinical samples we have constructed a set of monoclonal antibodies (MCAs) by immunisation with synthetic peptides (Evan *et al.*, 1985). The DNA sequence of the c-*myc* gene was used to predict the amino acid structure of the oncoprotein. Two peptides of 18 and 32 amino acids in length were synthesised. The regions chosen for synthesis and immunisation were predicted to be exposed within the intact molecule by assessing the relative hydrophobicity of different parts of the sequence. Six MCAs were produced that reacted to the 62,000 dalton protein identifiable with the c-*myc* product ($p62^{c\text{-}myc}$) (Evan *et al.*, 1985).

A possible tool in the detection and localisation of tumours is a suitably labelled tumour specific antibody. Several studies have shown that both polyclonal and monoclonal antibodies against cell surface antigens can effectively localise tumours (Mach *et al.*, 1981; Smedley *et al.*, 1983). However, the images obtained could be improved by the use of more tumour specific reagents. After labelling with radioiodine, one anti c-*myc* gene product antibody was found to localise human tumour xenografts in immunosuppressed mice. This antibody was therefore evaluated for its ability to localise bronchial carcinoma in patients.

Correspondence: S.Y.T. Chan.
*Present address: Christie Hospital & Holt Radium Institute, Manchester M20 9BX, UK.
Received 5 February 1986; and in revised form 2 June 1986.

© The Macmillan Press Ltd., 1986

### Materials and methods

### *Monoclonal antibody production*

Peptide synthesis, the immunisation protocol and screening procedures for deriving CT14 MCA (an IgG1κ) are described elsewhere (Evan *et al.*, 1985). Hybridoma cells were grown in the ascites fluid of female BALB/c mice. The antibody was purified by octanoic acid precipitation followed by ammonium sulphate concentration. Purified antibody was stored in PBS with 0.001% sodium azide, aliquoted and stored at −20°C. A non-specific monoclonal mouse immunoglobulin IgG1κ was used as a control prepared in the same manner.

### *Iodination*

Five mg of purified antibody in a total volume of PBS of 50 μl was added to an LP3 tube. Ten μl of chloramine T (BDH) at a concentration of $1\,mg\,ml^{-1}$ in fresh distilled water was added. Immediately 5 mCi of $^{131}I$ iodide ($200\,mCi\,ml^{-1}$, Amersham International) was added in a fume cupboard. The tube was stoppered and incubated at room temperature for 2 min. After this time, 50 μl of saturated tyrosine was added to terminate the reaction. A biogel P10 column was used to separate iodinated antibody from free iodine. Iodinated antibody was pooled and sterilized using a millipore 22 μm filter. This preparation was shown to localise xenografts of Colo-320 human apudoma cells growing in nude mice (Chan *et al.*, submitted). Such tumours are known to express considerably elevated levels of $p62^{c\text{-}myc}$.

### *ELISA assay*

Affinity of the labelled and unlabelled CT14 MCA to the synthetic peptide used for their production was compared in an ELISA assay. Five pmoles of the peptide in 100 μl of 0.1 M sodium bicarbonate buffer (pH 9.6) was added to each well of an 'Immulon 2' microtitre plate (Dynatech, Ltd.) for overnight incubation at room temperature. Unabsorbed peptides were removed by washing with TBS (25 mM Tris HCl 144 mM NaCl pH 8.1). This was followed by two 30 min incubations at room temperature with serial dilution of CT14 MCA in TBS containing 10% foetal calf serum; and then 100 μl per well of 1:150 dilution of rabbit anti-mouse Ig-horseradish peroxidase conjugate (Dako Ltd.), with washing in TBS between the steps. Quantitation of the bound CT14 MCA was provided by colour change on addition of the enzyme substrate (1 mM ABTS (Boehringer Mannheim Ltd.) in 0.1 M sodium citrate pH 5.0 and 1:1000 of 30% $H_2O_2$) and incubated in the dark for 30 min. Optical density of each well was measured by spectrophotometry at 406 mM using an ELISA plate reader.

### *Clinical studies*

Informed consent was obtained from all patients. Potassium iodide tablets (120 mg daily) were given orally for 2 days, from the day of antibody injection, to block thyroid uptake of radioiodine. One mg of CT14 labelled with 1 mCi $^{131}I$ in 10 ml normal saline was injected i.v. Serum samples in 6 patients were collected at 0, 1, 2, 4, 8, 24, 48 h after the administration of CT14. The samples were counted for $^{131}I$ activity both before and after TCA precipitation to determine protein bound and free $^{131}I$. Scanning using a gamma camera (IGE480) was performed at 24 h and 48 h after antibody injection. Thirty minutes prior to each scan patients were given 0.5 mCi of a standard technetium labelled human serum albumin (HSA) i.v. to delineate the blood pool.

Images of the distribution of HSA and CT14 were recorded on a Nodecrest computer which simultaneously recorded the energy peaks of $^{99M}Tc$ and $^{131}I$ respectively. The ratio of $^{131}I$ and $^{99M}Tc$ counts over the ventricular chambers, which is predominantly blood pool in nature, provides the standard for correction of circulatory antibody. The computer subtracts the $^{131}I$ image, pixel by pixel using this standard and produces an image which represents, by means of colour variation, areas of high concentration of bound $^{131}I$ antibody. Twenty patients with histologically proven cancer were studied: 14 with primary bronchial carcinoma; 4 with pulmonary metastases from tumours at other sites and 2 with localised primary tumours at sites outside the thorax.

### Results

Results of the ELISA assay (Figure 1) showed that the 131-iodine labelled CT14 MCA has retained its affinity to the synthetic peptide. Figure 2 shows the serum levels of $^{131}I$, measured as radioactivity per ml of blood detected by a gamma counter. Trichloroacetic acid precipitation showed that 90% of the $^{131}I$ remained protein bound. The mean half life of CT14-$^{131}I$ was 20 h and the slope of decay similar in all patients studied.

Good tumour localisation was seen in 12 out of 14 patients with primary bronchial carcinoma (Table I). Examples of the scans and radiological investigations are shown in Figures 3–7. All 12 patients had primary tumours of greater than 3 cm diameter. The 2 patients who gave no evidence of tumour localisation had smaller tumours – less than 2 cm diameter. Several patients giving good primary

**Figure 1** ELISA assay of CT14 anti $p62^{c-myc}$ activity before (○) and after (△) iodination.

**Figure 2** Serum levels of $^{131}$I-CT14 after i.v. injection into 6 patients.

**Table I** Patients with primary bronchial carcinoma.

| *No.* | *Age* | *Sex* | *Histology* | *CT14-$^{131}$I scan of chest* |
|---|---|---|---|---|
| 1 | 64 | M | SCLC | + |
| 2 | 55 | M | SCLC | + |
| 3 | 61 | M | SCLC | + |
| 4 | 64 | F | SCLC | + |
| 5 | 60 | M | SCLC | + |
| 6 | 72 | M | SCLC | + |
| 7 | 65 | F | SCLC | + |
| 8 | 48 | M | Squamous | + |
| 9 | 73 | M | Squamous | − |
| 10 | 72 | M | Squamous | + |
| 11 | 69 | M | Adeno | + |
| 12 | 64 | F | Adeno | − |
| 13 | 48 | M | Adeno | + |
| 14 | 60 | M | LCLC | + |

SCLC = Small cell lung cancer and LCLC = Anaplastic large cell lung cancer.

**Table II** Patients with preliminary metastases.

| *No.* | *Age* | *Sex* | *Primary tumours* | *CT14-$^{131}$I scan of chest* |
|---|---|---|---|---|
| 15 | 38 | M | Malignant melanoma | — |
| 16 | 32 | F | Breast cancer | — |
| 17 | 62 | F | Colonic cancer | — |
| 18 | 80 | M | Colonic cancer | — |
| 19 | 49 | M | Glioma | — |
| 20 | 70 | M | Rectal cancer | — |

tumour images had documented evidence of metastatic disease in liver, bone and brain. However, there was no evidence of increased uptake at the sites of metastases.

None of the 6 patients with pulmonary metastases showed tumour localisation. These patients all had multiple small metastases each less than 2 cm diameter (Table II). A patient with a primary glioma and another with locally recurrent rectal carcinoma gave no evidence of increased uptake within the thorax.

A control study was performed using 1 mg of non-specific mouse IgG labelled with 1 mCi $^{131}$I. Figure 4d shows the image obtained in a control scan in patient 5 whose primary lung tumour clearly demonstrated increased uptake of CT14-$^{131}$I. This scan was performed 7 days after the administration of labelled MCA. No evidence of tumour uptake was observed.

**Figure 3** Patient 5 – Large left upper lobe tumour. (a) Chest X-ray; (b) subtraction scan; (c) composite of scanning data showing $^{131}$I counts, $^{99M}$Tc counts and subtraction, 48 h after injection of MCA.

**Figure 4** Patient 5 scanned with control IgG showing no tumour uptake either on primary scan or after subtraction 48 h after injection.

### Discussion

We believe this to be the first report of tumour localisation by a monoclonal antibody against an oncogene product. In the majority of patients with primary bronchial carcinoma good tumour localisation corresponding to the chest X-ray and CT scan images was seen. There is good evidence from immunoprecipitation and immunoblotting data with fresh tumour lysates that CT14 binds specifically to $p62^{c\text{-}myc}$ (Sikora *et al.*, 1986). Furthermore, high levels of c-*myc* RNA have been reported in primary bronchial carcinomas indicating that the c-*myc* oncoprotein is likely to be abundant (Slamon *et al.*, 1984).

The c-*myc* protein is normally associated with cell nuclei. From this site that it is thought to exert a controlling function on cell division and differentiation. In large primary tumours, cell death will release nuclear contents into the surrounding extracellular space. It is presumably here that $p62^{c\text{-}myc}$ is being detected by radiolabelled MCA. Patients with small primary or metastatic tumours showed no tumour localisation. This may well be due to lack of sufficient tumour necrosis to significantly increase the extracellular $p62^{c\text{-}myc}$ level to detectable levels.

Because of their low sensitivity, it is unlikely that such scans will be useful in detecting disease not visualised by conventional radiological means. However, the level of $p62^{c\text{-}myc}$ in and around a tumour may provide a new biological pointer to tumour behaviour and response to therapy. The prompt disappearance of $p62^{c\text{-}myc}$ image shortly after chemotherapy may be the earliest indication of tumour response. Furthermore, the shedding of this oncoprotein by tumour cells into the blood stream could well provide a new marker of tumour load. The development of a suitable assay to test this hypothesis is currently in progress.

**Figure 5** Patient 7. (a) Chest X-ray showing left hilar carcinoma with disease in ipsilateral pleura; (b) CT scan from same patient; (c) subtraction scan: (d) composite as **Figure 3** – 48 h after MCA.

**Figure 6** Patient 11 showing tumour in right upper lobe. X-ray and scans as for Patient 5 (**Figure 3**).

**Figure 7** Patient 2. (a) Chest X-ray showing tumour in right mid and lower zones; (b) CT scan; (c) unsubtracted MCA image at 48 h.

### References

BISHOP, J.M. (1983). Cancer genes come of age. *Cell*, **4**, 1018.

CHAN, Y.T., EVAN, G. & SIKORA, K. (1986). Localisation of human tumour xenografts by radiolabelled monoclonal antibodies against the c-*myc* oncogene product. (Submitted).

COOPER, G.M. & LANE, M.A. (1984). Cellular transforming genes and oncogenesis. *Biochem. Biophys. Acta* **738**, 9.

DER, C.J. & COOPER, G.M. (1983). Altered gene products are associated with activation of cellular *ras*$^{k}$ genes in human lung and colon carcinomas. *Cell*, **32**, 201.

DOWNWARD, J, YARDEM, Y, MAYES, E. & 6 others (1984). Close similarities of epidermal growth factor receptor and v-erb-B oncogene protein sequences. *Nature*, **307**, 521.

EVAN, G., LEWIS, G.K., RAMSAY, G. & BISHOP, J.M. (1985). Isolation of monoclonal antibodies specific for the human and mouse c-*myc* proto-oncogene products. *Mol. Cell. Biol.*, **5**, 3610.

KELLY, K., COCHRAN, B.H., STILES, C.D. & LEDER, P. (1983). Cell specific regulation of the c-*myc* gene by lymphocyte mitogens and platelet derived growth factor. *Cell*, **35**, 603.

KRONTIRIS, T.G. (1983). The emerging genetics of human cancer. *New Engl. J. Med.*, **309**, 404.

LITTLE, C.D., NAU, M.M., CARNEY, D.N., GAZDAR, A.F. & MINNA, J.D. (1983). Amplification and expression of the c-*myc* oncogene in human lung cancer cell lines. *Nature*, **306**, 194.

MACH, J.P., CARRELL, S., FORNI, M., RITSCHARD, J., DONATH, A. & ALBERTO, P. (1981). Uses of radiolabelled monoclonal antibody against CEA for the detection of human carcinomas by external photoscanning and tomoscintigraphy. *Immunology Today*, **2**, 239.

PAUZA, D., & EVAN, G. (1986). Accumulation of p62$^{c-myc}$ in mitogen stimulated human lymphocytes. *Mol. Cell. Biol.*, (in press).

PFEIFER-OHLSSON, S., GOUSTIN, A.S., RYDNERT, J. & 4 others (1984). Spatial and temporal pattern of cellular *myc* oncogene expression developing human placenta: Implications for embryonic cell proliferation. *Cell*, **38**, 585.

RABBITTS, P.H., LAMOND, A., WATSON, J.V. & 4 others (1985). c-*myc* mRNA and protein metabolism in the cell cycle. *Embo. J.*, **4**, 2009.

SIKORA, K., CHAN, S., EVAN, G., MARKHAM, N. & STEWART, J. (1986). c-*myc* oncogene expression in colorectal cancer. (Submitted).

SLAMON, D.J., DEKERNION, J.B., VERMA, I.M. & CLINE, M.J. (1984). Expression of cellular oncogenes in human malignancies. *Science*, **224**, 256.

SMEDLEY, H.M., FINAN, P., LENNOX, E. & 4 others (1983). Localisation of metastatic carcinoma by a radiolabelled monoclonal antibody. *Br. J. Cancer*, **47**, 253.

SOUHAMI, R.L. (1984). The management of advanced non-small cell carcinoma of the bronchus. In *The Management of Lung Cancer*, Smyth J.F. (ed.). Edward Arnold Ltd., p. 132.

WATERFIELD, M.D., SCRACE, G.T., WHITTLE, N. & 7 others (1983). Platelet derived growth factor is structurally related to the putative transforming protein p28$^{sis}$ of simian sarcoma virus. *Nature*, **304**, 35.